# Prognostic implications of unrecognized myocardial infarction before elective percutaneous coronary intervention

**DOI:** 10.1038/s41598-022-26088-z

**Published:** 2022-12-14

**Authors:** Kai Nogami, Masahiro Hoshino, Yoshihisa Kanaji, Tomoyo Sugiyama, Toru Misawa, Masahiro Hada, Masao Yamaguchi, Tatsuhiro Nagamine, Yun Teng, Hiroki Ueno, Kazuki Matsuda, Kodai Sayama, Tsunekazu Kakuta

**Affiliations:** Division of Cardiovascular Medicine, Tsuchiura Kyodo General Hospital, 4-1-1 Otsuno, Tsuchiura City, Ibaraki 300-0028 Japan

**Keywords:** Interventional cardiology, Coronary artery disease and stable angina

## Abstract

Unrecognized myocardial infarction (UMI) is associated with adverse outcomes. This prospective, single-center study elucidated the prevalence and prognostic significance of UMI before elective percutaneous coronary intervention (PCI) using delayed-enhancement cardiac magnetic resonance (DE-CMR). We enrolled 236 patients with stable coronary artery disease who underwent DE-CMR before elective PCI. The prevalence of UMI and the association of clinical and CMR-derived variables with major adverse cardiac events (MACE), defined as cardiovascular death, nonfatal MI, hospitalization for congestive heart failure, and unplanned late revascularization, were assessed. Final analysis revealed that 63/213 (29.6%) patients had UMI. Target territory UMI was observed in 38 patients (17.8% of the total cohort, 60.3% of patients with UMI). UMI was significantly associated with sex, diabetes mellitus, left ventricular ejection fraction, SYNTAX score, and fractional flow reserve in the target vessels. During follow-up (median, 23 months), MACE occurred in 17 (27.0%) patients with UMI and 17 (11.3%) without UMI (*P* = 0.001). Multivariable modeling revealed that UMI (hazard ratio: 2.18, 95%CI, 1.10–4.33, *P* = 0.001) was an independent predictor of MACE. Kaplan–Meier analysis indicated that the presence of UMI was significantly associated with a higher incidence of MACE. UMI was independently associated with a greater risk of MACE after successful PCI.

## Introduction

A substantial proportion of acute myocardial infarction (MI) is asymptomatic or atypical in presentation, which escapes clinical identification^1,2^. The prevalence of unrecognized myocardial infarction (UMI) varies, depending on the study population and the modality used for investigation. The reported prevalence of UMI is up to 30% in patients with suspected coronary artery disease (CAD)^3,4^. Previous investigations have suggested that the presence of UMI increases the risk of adverse events^1,2,5^. The association between the functional severity of ischemia, represented by fractional flow reserve (FFR), and the occurrence of UMI in the myocardial segment supplied by the coronary artery in question or the impact of UMI on the patients’ outcomes has not been investigated in patients undergoing elective percutaneous coronary intervention (PCI). FFR represents the severity of ischemia in the studied territory and the prevailing hypothesis posits that myocardial ischemia is the primary risk factor linked to poor prognosis in patients with CAD^6,7^. Therefore, the aims of the present study were two-fold: (1) to investigate the prevalence of UMI detected by gadolinium contrast-based delayed-enhancement cardiac magnetic resonance (DE-CMR) imaging in patients with stable CAD without a known history of MI or revascularization, who were scheduled to undergo elective PCI; and (2) to test the hypothesis that UMI is associated with adverse outcomes after successful PCI.

## Methods

### Study design and patient population

**Figure 1:**
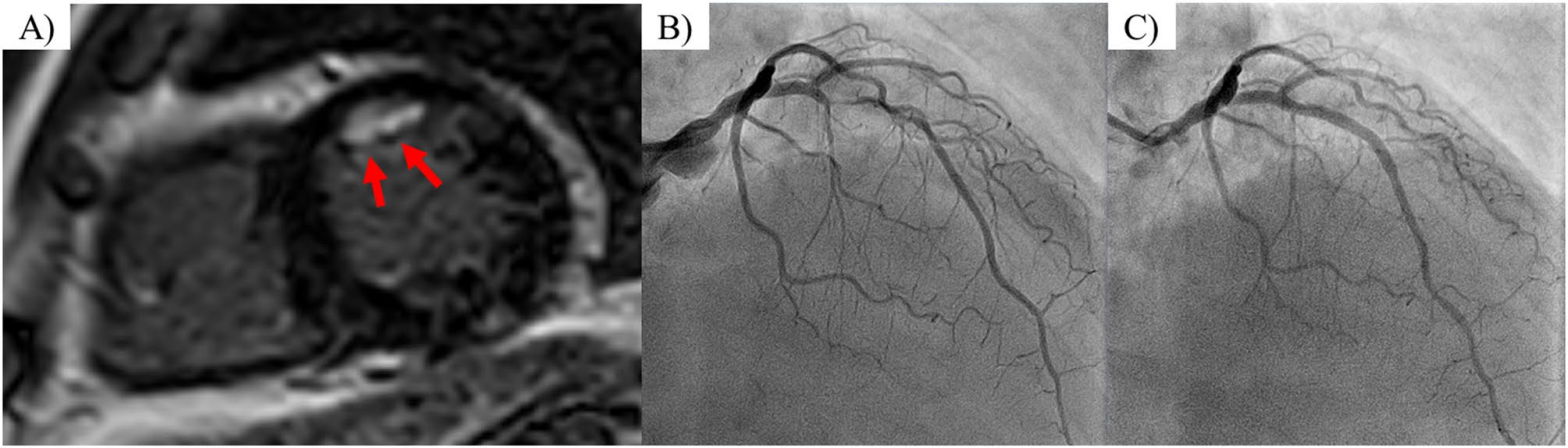
Representative images of patients with unrecognized myocardial infarction. A 69-year-old man with a history of diabetes mellitus with stable coronary artery disease. (**A**) Short-axis view of delayed-enhancement cardiac magnetic resonance imaging with anterior late gadolinium enhancement (arrowheads). (**B**) Pre-PCI angiography showing significant coronary stenosis in the proximal left anterior descending artery. The FFR value was 0.53. (**C**) Post-PCI angiography demonstrating implantation of a drug-eluting coronary stent in the LAD lesion. The FFR value after PCI was 0.91. *PCI* percutaneous coronary intervention, *FFR* fractional flow reserve, *LAD* left anterior descending artery.

This prospective study was conducted at Tsuchiura Kyodo General Hospital between November 2017 and April 2020. We prospectively enrolled patients with stable CAD (N = 236) who were scheduled to undergo elective PCI. Preprocedural DE-CMR evaluation was performed according to the standard protocol. These patients were selectively chosen from our regular clinical population based on the following inclusion criteria: age > 20 years and detection of an identifiable, de novo single culprit lesion located in the proximal portion of a native coronary artery; and symptomatic or objective ischemia detected by noninvasive stress testing, and/or FFR measurements. Stable CAD was defined as the occurrence of angina symptoms of consistent frequency, duration, and intensity within 6 weeks before PCI. The target lesion was identified based on the combination of diagnostic coronary angiography, angiographic lesion morphology, electrocardiography findings, noninvasive stress testing, or FFR values. The exclusion criteria included a past history of MI, angiographically significant left main disease, past history of PCI or coronary artery bypass surgery, renal insufficiency with baseline serum creatinine level > 1.5 mg/dL, cardiomyopathy, and congestive heart failure. Patients with impaired systolic function (< 50%) were also excluded. After PCI, we excluded patients with periprocedural MI as defined by the Fourth Universal Definition of Myocardial Infarction^8^, based on a blood sample obtained after an average of 20–24 h, symptoms, and other objective findings after PCI completion, because these events can affect the prognosis. Patients with minor cardiac troponin elevation, and those without other manifestations established by the above-mentioned definition, were included in the final analysis. Figure 1 depicts a representative case of a patient undergoing FFR-guided PCI with preprocedural physiological assessment using DE-CMR. This study was conducted in compliance with the guidelines and approval (TKGH-IRB 2017 # 628) of the Institutional Ethics Committee of Tsuchiura Kyodo General Hospital. This study also complied with the Declaration of Helsinki for investigation in humans. All patients provided written informed consent before enrollment.

### Invasive coronary angiography, intracoronary physiological assessment, and percutaneous coronary intervention

Additional procedure details are presented in the Supplementary Methods. Angiographically intermediate lesions were physiologically assessed using FFR. After wire calibration, the intracoronary pressure distal to the coronary stenosis was measured. Subsequently, FFR was calculated as the ratio of the mean distal coronary to aortic pressure during maximum hyperemia, which was induced by intravenous adenosine infusion (140 μg/kg per min through a central vein). All patients were instructed to strictly refrain from ingesting caffeinated beverages for > 24 h before catheterization. Eligible patients were subsequently scheduled for pre-PCI CMR (median 11 days after diagnostic catheterization, range: 3–16 days) and elective PCI (median 5 days from CMR, range: 2–7 days). The PCI procedure was performed via the radial artery using a 6-F system. All patients underwent coronary stent implantation (drug-eluting stent, 100%) with predilatation. Successful PCI was defined as the presence of < 20% residual stenosis, with thrombolysis in myocardial infarction grade 3 flow, no side branch occlusion (> 2 mm in diameter) or distal embolization, and the absence of periprocedural MI (Type 4A). Pharmacotherapy was initiated for all patients promptly after enrollment.

### CMR examination

#### CMR acquisition and cine-CMR

The additional acquisition details are presented in the Supplementary Methods. Imaging was performed before PCI using a 1.5-T scanner (Philips Achieva, Philips Medical Systems, Best, the Netherlands) with 32-channel cardiac coils. Cine-CMR was performed using a retrospectively gated steady-state free-precession sequence. Twelve short-axis slices of the left ventricle were acquired from the apex to the base. After cine-CMR imaging acquisition, a gadolinium-based contrast agent was infused intravenously at a total dose of 0.10 mmol/kg. Fifteen minutes after injection, late gadolinium enhancement (LGE) imaging was acquired along the same planes as cine imaging. UMI was defined as the absence of a history of MI and/or revascularization in the medical records, but the detection of LGE by consensus of two experienced cardiologists (K.N. and Y.K.) blinded to the patient data. LGE was assessed on the short-axis images using manual planimetry. A reference region of interest (ROI) was placed in the remote myocardium for objective quantification of LGE. A myocardial region was considered to be affected if at least 10 adjacent myocardial pixels revealed a signal intensity of 5 standard deviations above the mean intensity of the reference ROI. A high-signal intensity lesion located in the subendocardial territories consistent with specific epicardial coronary arteries on LGE images was considered to be representative of UMI. UMI mass was calculated by multiplying the volume by the density of the myocardial tissue (1.05 g/mL).

#### Assessment of outcomes

Patients were followed-up to determine the occurrence of the predefined primary endpoint, major adverse cardiovascular events (MACE), i.e., cardiovascular death, nonfatal MI, hospitalization for congestive heart failure, unplanned late revascularization (more than 3 months after PCI), and ischemic stroke. Patients’ outcomes were also assessed using the exploratory MACE endpoint: cardiovascular death, nonfatal MI, hospitalization for congestive heart failure, and stroke. Clinical endpoints were determined by blinded assessment of hospital records or via telephone interviews. The time to event was calculated as the period between PCI and the first occurrence of MACE. The data of patients without MACE were censored at the time of the last follow-up.

#### Sample size estimation

The estimated sample size was calculated based on the following assumptions: (1) 30% prevalence of UMI in patients with CAD^4^, (2) increase in the hazard ratio to 2.6 for patients with UMI^9^, and (3) 1-year risk of 7.0% for the primary endpoint (MACE)^10^. These assumptions imply an event rate of 12.3% and 4.7% in patients with and without UMI for this cohort during the 1-year follow-up, respectively. A total of 201 patients would be required to evaluate a statistically significant difference at the 5% (two-sided test) level with a power of 80%. Thus, we, prospectively enrolled 236 patients to test our hypothesis, considering a 15% exclusion rate for the final analysis due to unsatisfactory CMR imaging or loss to follow-up.

### Statistical analysis

Participants were first divided into two groups, i.e., the UMI and non-UMI groups, and their characteristics were compared to determine the predictors of UMI. Thereafter, the clinical characteristics and CMR-derived variables were compared between the groups with or without MACE. Statistical analyses were performed using SPSS version 25.0 (IBM Corporation, Armonk, NY, USA) and R version 4.0.3 (The R Foundation for Statistical Computing, Vienna, Austria) software. Categorical data were expressed as numbers and percentages and compared using the chi-squared or Fisher’s exact test. Continuous data were expressed as the median (interquartile range) and analyzed using the Mann–Whitney U test. The analysis of variance was used for variables with non-normal and normal distribution to evaluate the differences between the groups with and without MACE, respectively. Correlations between two variables were assessed using Pearson’s correlation analysis. Receiver operating curves were analyzed to assess the best cut-off values of the LGE mass for predicting MACE. The optimal cut-off value was calculated using the Youden index. Survival curves were estimated using Kaplan–Meier analysis and compared using log-rank tests. A Cox proportional hazards regression model was constructed to identify independent predictors of MACE. Covariates with *P* < 0.05 in the univariate analysis were included in the multivariate analysis. The collinearity index was used to check linear combinations among covariates, and backward stepwise selection using the p-value to avoid overfitting. A prediction model was constructed for MACE to determine the incremental discriminatory and reclassification performance by adding UMI to the clinical model using the relative integrated discrimination improvement and category-free net reclassification index. UMI was added to the clinical risk model, which included glycated hemoglobin (HbA1c), dyslipidemia, and the Synergy Between percutaneous coronary intervention With Taxus and coronary artery bypass surgery (SYNTAX) score. Two-sided *P* values < 0.05 were considered statistically significant.

## Results

### Baseline patient characteristics, angiographical, physiological and CMR findings

**Figure 2:**
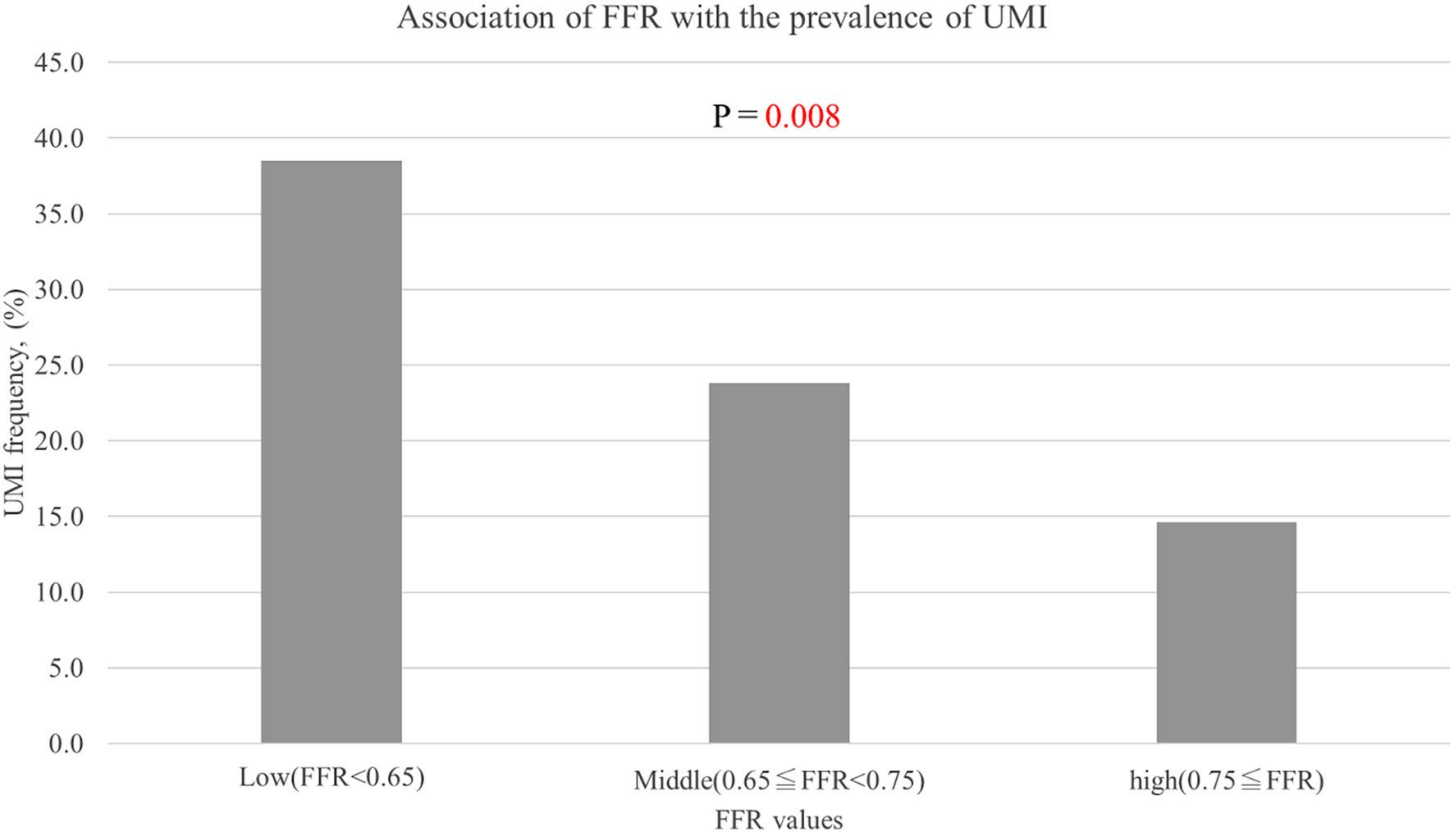
Association of FFR with the prevalence of unrecognized myocardial infarction. When patients were divided into tertiles based of FFR of culprit lesion stenosis, the frequency of UMI was significantly higher in the lower FFR group. *FFR* fractional flow reserve, *UMI* unrecognized myocardial infarction.

Six of 236 patients were excluded because of the inability to cross the lesion with the pressure wire or suboptimal quality of physiological data acquisition. Additionally, 7 patients were also from the final analysis because they developed periprocedural MI due to side branch occlusion or distal embolization (Type 4A MI). Ten patients were further excluded due to failure to obtain satisfactory CMR data. Thus, the final analysis included 213 patients with complete pre-PCI regional physiological and DE-CMR data. UMI was detected in 63 patients (29.6%). FFR could be measured in 192/213 patients (90%). The patients’ baseline characteristics, physiological findings, and CMR data stratified according to the presence or absence of UMI are presented in Table 1. Patients with UMI were more likely to be men, and have higher SYNTAX scores, lower ejection fraction (EF), higher N-terminal-pro B-type natriuretic peptide levels, and a higher prevalence of diabetes mellitus compared with those without UMI. Patients with UMI had greater high-sensitivity troponin I levels at presentation compared to those without UMI [12 (7–30) ng/L vs 6 (3–17) ng/L, *P* = 0.002; Table 1]. No significant relationship was observed between elevation in high-sensitivity cardiac troponin I (hs-cTnI) after PCI and the presence of UMI. UMI was located significantly more frequently in the target vessel territories of PCI [38/63 (60.3%) vs 27/126 (21.4%), *P* < 0.001]. The proportion of UMI in the target vessel territories of PCI did not differ significantly among the right coronary, left anterior descending, and left circumflex arteries (11.5%,12.9%, and 4.8% respectively; *P* = 0.714). The size of the UMI did not differ significantly among the three coronary arteries (median size of the UMI: 11.2 g, 7.2 g, and 13.0 g for the right coronary, left anterior descending, and left circumflex arteries, respectively; *P* = 0.23). Although multi-segment UMI was observed in 9 patients, the risk factors did not differ significantly between these patients and those with a single UMI. Overall, the presence of UMI was significantly associated with the severity of FFR, according to previously reported cutoff values^11,12^ (Fig. 2).

**Table 1:** Baseline patient characteristics.

	All (n = 213)	Patients without UMI (n = 150)	Patients with UMI (n = 63)	*P* value
**Baseline data**				
**Demographics**				
Sex				< 0.001
Men, n (%)	164 (77.0)	105 (70.0)	59 (93.7)	
Women, n (%)	49 (23.0)	45 (30.0)	4 (6.3)	
Age, years	68 [61, 72]	68 [62, 73]	66 [60, 71]	0.143
Body surface area, m^2^	1.72 [1.58, 1.82]	1.66 [1.54, 1.82]	1.77 [1.71, 1.84]	0.001
Body mass index, kg m^−2^	24.4 [22.2, 27.0]	24.0 [21.9, 26.4]	25.4 [23.2, 27.5]	0.008
Rate pressure product at rest	9432 [8100, 10725]	9432 [8012, 10721]	9432 [8320, 10590]	0.953
Rate pressure product at hyperemia	10,108 [8585, 11616]	10,077 [8623, 11775]	10,240 [8602, 11006]	0.627
**Medical history**				
Current smoker, n (%)	53 (24.9)	32 (21.3)	21 (33.3)	0.082
Diabetes mellitus, n (%)	90 (42.3)	55 (36.7)	35 (55.6)	0.015
Hypertension, n (%)	157 (73.7)	108 (72.0)	49 (77.8)	0.495
Hyperlipidemia, n (%)	111 (52.1)	82 (54.7)	29 (46.0)	0.293
Family history, n (%)	28 (13.1)	21 (14.0)	7 (11.1)	0.661
**Laboratory data**				
Creatinine, mg dL^−1^	0.83 [0.72, 0.95]	0.81 [0.70, 0.94]	0.85 [0.78, 1.00]	0.009
eGFR, ml min^−1^ 1.73 m^−2^	68.6 [58.8, 77.7]	68.3 [59.4, 77.0]	70.9 [54.4, 77.8]	0.786
LDL-C, mg dL^−1^	97 [75, 120]	97 [75, 121]	94 [79, 113]	0.511
HDL-C, mg dL^−1^	51 [43, 60]	51 [44, 60]	48 [40, 58]	0.083
Triglyceride, mg/dL	122 [87, 165]	121 [86, 165]	128 [88, 165]	0.767
HbA1c, %	6.1 [5.7, 6.9]	6.0 [5.6, 6.8]	6.4 [5.8, 7.6]	0.007
NT-pro BNP, ng L^−1^	128 [52, 291]	96 [44, 221]	196 [85, 570]	0.006
hs-cTnI at presentation, ng L^−1^	9 [3, 26]	6 [3, 17]	12 [7, 30]	0.002
**Angiographic characteristics**				
Lesion location (LAD/LCX/RCA)	140/21/52	101/15/34	39/6/18	0.675
Reference diameter, mm	2.44 [1.98, 2.78]	2.47 [2.06, 2.79]	2.24 [1.82, 2.76]	0.111
Minimum lumen diameter, mm	0.83 [0.58, 1.14]	0.88 [0.65, 1.19]	0.72 [0.30, 1.01]	0.004
Angiographic stenosis severity, %	64.9 [53.1, 76.5]	61.9 [52.9, 73.7]	71.0 [57.9, 86.0]	0.004
SYNTAX score	10.0 [7.0, 15.0]	9.0 [7.0, 12.8]	14.5 [8.0, 21.3]	< 0.001
pre FFR	0.64 [0.48, 0.73]	0.68 [0.54, 0.75]	0.51 [0.27, 0.69]	< 0.001
**CMR analysis**				
LV end-diastolic volume, ml	116 [96, 140]	109 [94, 131]	136 [113, 175]	< 0.001
LV end-systolic volume, ml	41 [31, 60]	36 [30, 48]	62 [40, 93]	< 0.001
LVEF, %	65 [60, 69]	67 [63, 72]	58 [54, 65]	< 0.001
LV mass, g	135 [109, 160]	125 [103, 146]	154 [137, 191]	< 0.001
LV mass index, g m^−2^	76.5 [65.3, 92.9]	73.3 [63.8, 84.7]	85.7 [73.2, 109.4]	< 0.001
**Post PCI data**				
Peak hs-cTnI, ng L^−1^	229 [82, 1118]	235 [89, 1099]	188 [71, 1284]	0.512
Peak CK, IU L^−1^	104 [73, 187]	104 [73, 178]	102 [70, 200]	0.868
Peak CK-MB, IU L^−1^	12 [9, 19]	12 [10, 18]	12 [8, 20]	0.602
Post FFR	0.87 [0.82, 0.92]	0.87 [0.83, 0.92]	0.84 [0.77, 0.92]	0.188

Data are presented as number (%), median (interquartile range).

*CK* creatine kinase, *EF* ejection fraction, *eGFR* estimated glomerular filtration rate, *FFR* fractional flow reserve, *HbA1c* glycosylated hemoglobin, *HDL-C* high-density lipoprotein cholesterol, *hs-cTnI* high-sensitivity cardiac troponin-I, *LAD* left anterior descending coronary artery, *LCx* left circumflex coronary artery, *LDL-C* low-density lipoprotein cholesterol, *LV* left ventricular, *NT-proBNP* N-terminal pro B-type natriuretic peptide, *RCA* right coronary artery, *UMI* unrecognized myocardial infarction.

### Determinants of the presence of UMI

Table 2 depicts the results of univariate and multivariate logistic regression analyses performed to determine the predictors of the presence of UMI. Sex, diabetes mellitus, EF, SYNTAX score, and pre-PCI FFR were independent predictors of the presence of UMI.

**Table 2:** Predictors of unrecognized myocardial infarction (Logistic regression analysis).

	Univariable			Multivariable		
	Hazard ratio	95% CI	*P* value	Hazard ratio	95% CI	*P* value
Men	6.32	(2.17–18.50)	< 0.001	4.94	(1.46–16.70)	0.010
Body Surface Area	13.70	(2.61–71.50)	0.002			
Body Mass Index	1.12	(1.03–1.22)	0.008			
Diabetes mellitus	2.16	(1.19–3.93)	0.012	2.53	(1.18–5.42)	0.017
HbA1c	1.54	(1.19–2.00)	0.001			
Creatinine	9.16	(1.94–43.30)	0.005			
NT-pro BNP	1.00	(1.00–1.00)	0.170			
hs-cTnI at presentation	1.01	(1.00–1.02)	0.092			
End diastolic LV volume	1.02	(1.01–1.03)	< 0.001			
End systolic LV volume	1.03	(1.01–1.04)	< 0.001			
LVEF	0.92	(0.89–0.95)	< 0.001	0.94	(0.91–0.97)	< 0.001
LV mass index	1.03	(1.02–1.05)	< 0.001			
Angiographic stenosis severity	1.03	(1.01–1.05)	0.002			
Minimum lumen diameter	0.36	(0.18–0.73)	0.004			
Total SYNTAX score	1.14	(1.08–1.20)	< 0.001	1.13	(1.07–1.20)	< 0.001
Pre FFR	0.01	(0.00–0.08)	< 0.001	0.01	(0.00–0.12)	< 0.001

Abbreviations are in Table 1. *CI* confidence interval.

### UMI and prognosis

The patients’ baseline characteristics, physiological findings, and CMR data stratified according to the presence or absence of primary MACE are presented in Supplementary Table S1. No patient was lost to follow-up. During the follow-up period of 23 months (18 months, 32 months), 34 patients reached the composite endpoint (primary outcome, i.e., MACE, 16.0%; Supplementary Table S2). Patients with MACE were more likely to have higher SYNTAX scores, a family history of CAD, and higher prevalence of diabetes mellitus and dyslipidemia compared to those without MACE.

**Figure 3:**
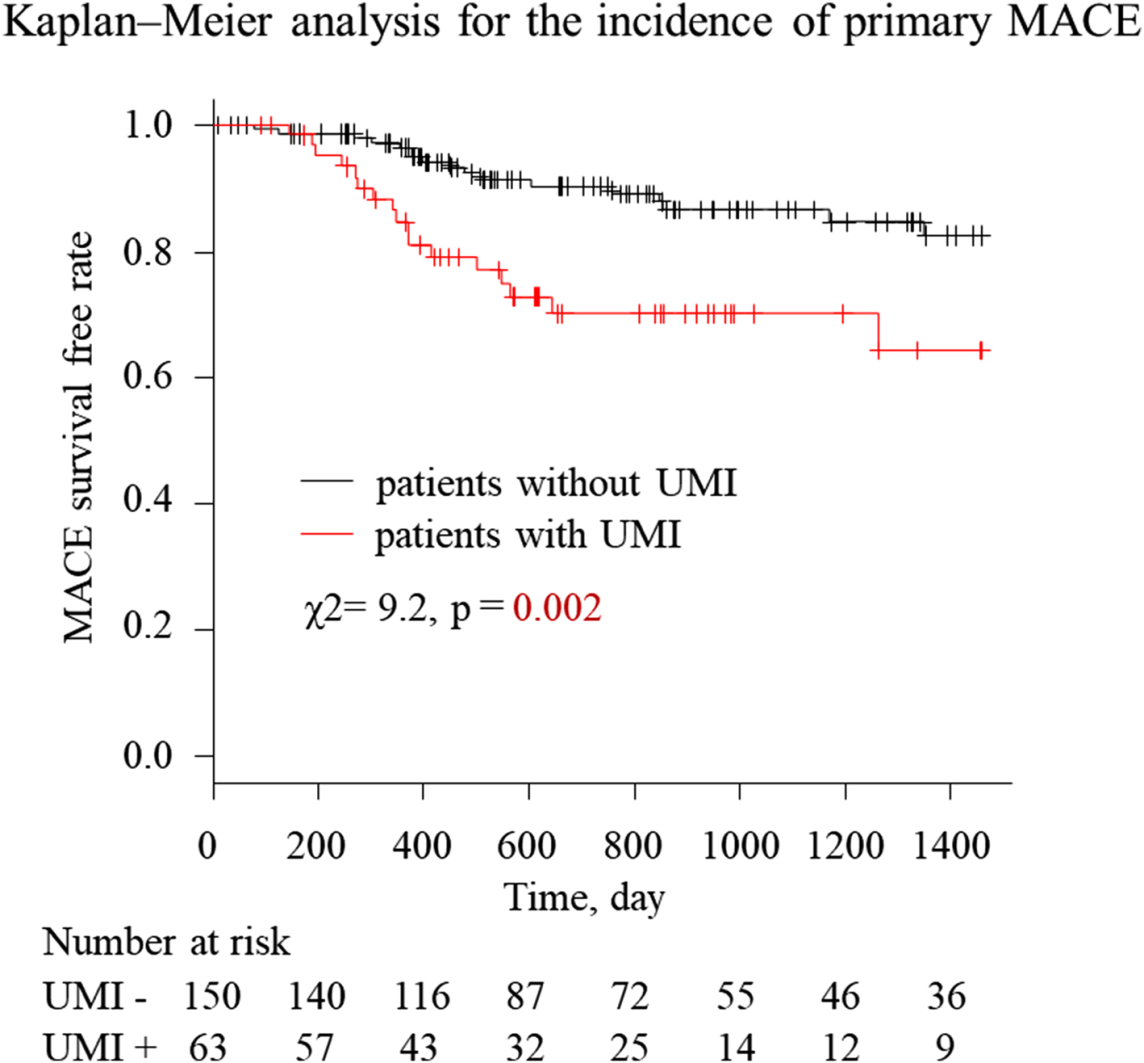
Kaplan–Meier analysis for the incidence of primary MACE according to the presence of UMI. The incidence of primary MACE was significantly higher in patients with UMI than that in those without UMI. Primary MACE was defined as cardiovascular death, nonfatal myocardial infarction, hospitalization for congestive heart failure, unplanned late revascularization and ischemic stroke. *UMI* unrecognized myocardial infarction, *MACE* major adverse cardiovascular events.

In the final analysis, 78 (36.6%) of 213 patients in showed elevation of hs-cTnI exceeding 5 times the upper reference limit based on blood samples obtained at an average of 21.0 ± 1.5 h after PCI completion. There was no significant association between post-PCI hs-cTnI elevation and MACE in the present study. No significant difference was observed in the prevalence of unplanned late revascularization between patients with and without UMI (9.3% vs 11.1%, *P* = 0.802). MI occurred significantly more often in patients with UMI (0.7% vs 7.9%, *P* = 0.009). The composite endpoint was reached in 27.0% (17/63) of patients with UMI reached a compared to 11.3% (17/150) of patients without UMI (total MACE, n = 34, odds ratio 2.87, 95% CI 1.27–6.55, *P* = 0.007). Cox proportional hazard analysis revealed that the presence of UMI and dyslipidemia were independent predictors of MACE (Table 3). Kaplan–Meier analysis indicated that the presence of UMI was significantly associated with a higher incidence of primary MACE (Fig. 3). When the composite endpoint was limited to cardiovascular death, nonfatal MI, heart failure hospitalization, and stroke (secondary endpoint of MACE), the presence of UMI and dyslipidemia remained significant and robust predictors of MACE (Table 3), although the number of adverse events was limited (n = 13) in this secondary exploratory endpoint analysis. Kaplan–Meier analysis also indicated that the presence of UMI was significantly associated with a higher incidence of secondary MACE (Supplementary Figure S1). Receiver operating curve analysis indicated the best cut-off values of the LGE mass for predicting primary and secondary MACE (primary MACE: 1.80 g, area under the curve: 0.619, *P* = 0.028; secondary MACE: 7.70 g, area under the curve 0.768, *P* = 0.001, respectively; Supplementary Figure S2). When patients were divided into two groups based on the best cut-off value of LGE mass, primary and secondary MACE occurred significantly more often in the larger LGE mass group (primary MACE 12.5% vs 24.6%, *P* = 0.038; secondary MACE 2.4% vs 19.6%, *P* < 0.001).

**Table 3:** Predictors of primary and secondary MACE (Cox proportional hazard model).

Predicting primary MACE	Univariable			Multivariable		
	HR	95% CI	*P* value	HR	95% CI	*P* value
Dyslipidemia	2.34	(1.12–4.91)	0.024	2.71	(1.29–5.70)	0.009
HbA1c	1.27	(1.06–1.51)	0.009			
UMI	2.72	(1.39–5.33)	0.004	3.10	(1.57–6.11)	0.001
LGE mass > 1.80	2.19	(1.11–4.32)	0.023			
LGE mass	1.04	(1.01–1.07)	0.006			
SYNTAX score	1.06	(1.01–1.10)	0.007			
Pre FFR	0.69	(0.10–4.62)	0.700			

Predicting secondary MACE	Univariable			Multivariable		
	HR	95% CI	*P* value	HR	95% CI	*P* value
Dyslipidemia	11.09	(1.44–85.31)	0.021	14.28	(1.85–110.30)	0.009
HbA1c	1.37	(1.06–1.76)	0.015			
UMI	8.41	(2.31–30.57)	0.001	10.42	(2.86–38.02)	0.003
LGE mass	1.07	(1.03–1.11)	< 0.001			
LGE mass > 7.70	9.17	(2.82–29.79)	< 0.001			
SYNTAX score	1.09	(1.03–1.16)	0.006			
Pre FFR	0.22	(0.01–4.34)	0.318			

Abbreviations are in Tables 1 and 2. LGE = late gadolinium enhancement; primary MACE was defined as cardiovascular death, nonfatal MI, hospitalization for congestive heart failure, unplanned late revascularization, and ischemic stroke. secondary MACE was defined as cardiovascular death, nonfatal MI, hospitalization for congestive heart failure, and ischemic stroke.

Reference prediction models were constructed to compare the discriminatory and reclassification ability of the presence of UMI for MACE (Table 4). The discriminatory ability was significantly improved by the addition of UMI to the model that included HbA1c, dyslipidemia, and SYNTAX score ≥ 23.

**Table 4:** Comparison of discriminant and reclassification capacities of each model for predicting primary MACE.

	C statistics	Relative IDI		Continuous NRI	
		Value	*P* value	Value	*P* value
Clinical model 1	0.673	Reference		Reference	
Clinical model 2	0.702	0.040	0.014	0.508	0.006

Two predictive models were constructed as follows: Clinical model 1 included HbA1c, dyslipidemia, SYNTAX score ≥ 23; and Clinical model 2 included a combination of Clinical model 1 and UMI.

## Discussion

Although DE-CMR is a highly accurate modality for detecting myocardial fibrosis and quantifying the size of the myocardial infarct, including small infarcts in patients with UMI, little is known about the prevalence of UMI in patients undergoing elective PCI and the prognostic significance of UMI. The present study is the first to demonstrate that the presence of UMI on DE-CMR can facilitate prognostication for patients undergoing elective PCI. The important findings of this study are as follows: (1) the prevalence of UMI was 29.6% in patients undergoing FFR-guided PCI; (2) the presence of UMI was associated with the FFR and angiographic stenosis severity, and 60.3% of UMI was located downstream of the target vessel for PCI; (3) UMI was found in angiographically normal vessel territory only in 1.6% (1/63) of the total cohort; (4) sex, diabetes mellitus, EF, SYNTAX score, and pre-PCI FFR were independently associated with the presence of UMI; and (5) the presence of UMI and dyslipidemia were independent predictors of MACE after successful PCI.

Our results demonstrated that CMR-detected UMI was not uncommon and provided significant prognostic information about conventional clinical risk factors, including sex, age, EF, angiographic information, pre-PCI FFR, angiographic data, and subclinical post-PCI cardiac marker elevation in patients undergoing elective PCI.

### Potential mechanisms causing UMI

The exact pathophysiological mechanisms underlying UMI remain to be elucidated. Our results are concurrent with those of previous studies, which demonstrated a significant association between angiographic stenosis severity and the presence of UMI^3^. This study extended the focus of research and demonstrated that the presence of UMI was significantly associated with functional stenosis severity, as represented by FFR. This observation is in agreement with a previous study that demonstrated the significant relationship between high-risk plaque features and FFR^13^. The progression of atherosclerosis leads to plaque instability, asymptomatic plaque rupture, and distal debris liberation accompanied by cardiac marker elevation, resulting in UMI in the subtended territory and stenosis progression in the lesion due to plaque healing, culminating in an increase in the plaque burden^13,14^. The occurrence of UMI in territories without significant atherosclerotic burden has been debated and remains unelucidated. Coronary spasm, microvascular disease, and type 2 MI may partially explain the occurrence of UMI^3,15–17^.

### Relationship between UMI and prognosis

In this study, the presence of UMI was suggestive of the presence of a severely advanced atherosclerotic burden. This notion was supported by the significant relationship between the angiographic and functional severity of epicardial stenosis and the SYNTAX score with the presence of UMI observed in the present study. Our results conform to those of previous studies^2,5,14,17,18^. When unplanned late revascularization was excluded from the primary composite MACE endpoint, UMI retained its significant association with a poor prognosis, which was designated as the secondary exploratory MACE endpoint (Table 3). Further studies are required to elucidate the mechanisms responsible for the relationship between UMI and poor prognosis. Previous studies have reported a significant relationship between UMI and the risk of heart failure^6,19^. This association may be linked to a worse prognosis in patients with UMI, irrespective of the presence or absence of ischemia or its severity^2^. Microvascular disease has been also postulated to be a mechanism underlying the link between UMI as a marker of advanced atherosclerosis and poor prognosis^20^. A population-based study found an association between UMI and cerebral infarction^21^. This finding suggests that UMI is a novel risk factor for cardiac embolism and cerebral infarction, although further studies are needed to test this hypothesis and whether anticoagulation therapy could be effective in reducing cerebral infarction in patients with UMI.

### Potential clinical implication

Consistent with previous studies, this study demonstrated that UMI confers a significantly higher risk of adverse events (although these lesions were small (median, 11.9 g) with preserved systolic function), independent of conventional risk factors, coronary anatomic disease burden, or the severity of ischemia represented by FFR, in patients undergoing elective PCI without a history of known MI or revascularization. Our results suggest that DE-CMR may identify patients at high risk for subsequent adverse events after elective and uncomplicated PCI, who cannot be identified using routine clinical workup, including angiography, pre-PCI FFR, EF, and pre- and post PCI cardiac marker elevation, although no specific therapeutic approach has been proposed for UMI at the moment. Our results further suggest that the presence of UMI may be a potentially important confounding factor when evaluating the benefit of an invasive approach compared to guideline-directed pharmacotherapy alone in randomized trials.

The current guidelines for secondary prevention programs have focused on nonfatal MI, death, and heart failure hospitalization as key risk factors and populations as candidates for the recommended treatment. Patients with UMI were precluded from the aggressive preventive management strategy and undertreated. Further prospective studies are needed to test whether early preventive intervention following the detection of UMI by DE-CMR could reduce subsequent risks. The results of the current and previous studies strongly imply the significant association between UMI and cardiovascular risk factors such as sex, diabetes mellitus, left ventricular mass index, EF, and coronary anatomy, although UMI remained as an independent risk factor for MACE after adjusting for conventional risk factors. Currently, a specific intervention or treatment that can improve the prognosis for patients with UMI after successful PCI remains elusive. Determining whether primary intense intervention for these conventional risk factors associated with the presence of UMI could reduce the development of UMI would be an important endeavor, since undetected silent ischemia is considered to be an important contributor to adverse events in patients with UMI^2,19^.

### Limitations

The findings of this should be interpreted in light of several important limitations. The present study evaluated a moderate number of patients using CMR but the sample population of patients with MACE was relatively small, resulting in the inherent limitations arising from the small size, single-center design, and observational nature; therefore, extensive planned subgroup analyses could not be performed and the possibility of selection bias cannot be eliminated. We did not include patients with metal device implantation, bronchospasm, claustrophobia, or atrioventricular block. This may have led to additional selection bias. However, we prospectively recruited patients from our regular clinical population and CMR scans were performed according to the research protocol, suggesting the reduction of referral bias. The assessment of myocardial segments affected by coronary stenosis was determined by the coronary anatomy, without employing an objective assessment method, although no universally accepted criteria exist for this purpose. The small number of events precludes the differentiation of “hard” endpoints, including cardiac death, which may be more important than emergent revascularization. Finally, the potentially poor symptom recognition in patients with UMI may also contribute to lower event rates during the follow-up period, although this hypothetical possibility may result in an increase in the risk in patients with UMI.

## Conclusion

UMI was not uncommon in prospectively enrolled patients scheduled to undergo elective PCI without a history of known MI and previous revascularization. DE-CMR provided significant incremental prognostic information, independent of conventional clinical risk factors, the SYNTAX score, post-PCI subclinical cardiac marker elevation, and pre-PCI FFR in the high-risk group.

## Supplementary Information

Supplementary Information.

## Data Availability

The data that support the findings of this study are available from the corresponding author on reasonable request.

## Abbreviations

CAD: Coronary artery disease
DE-CMR: Delayed enhancement cardiac magnetic resonance imaging
EF: Ejection fraction
FFR: Fractional flow reserve
LGE: Late gadolinium enhancement
MACE: Major adverse cardiovascular events
MI: Myocardial infarction
UMI: Unrecognized myocardial infarction
