# Supplementary material for: Prognostic implications of unrecognized myocardial infarction before elective percutaneous coronary intervention
Source: Sci Rep. 2022 Dec 14;12:21579. doi: 10.1038/s41598-022-26088-z (PMC9751065; doi:10.1038/s41598-022-26088-z)
Supplement: Supplementary file 1 — Supplementary Information. [file 41598_2022_26088_MOESM1_ESM.pdf]

Supplementary Methods

Invasive Coronary Angiography, Intracoronary Physiological Assessment and Percutaneous Coronary Intervention

Each patient initially underwent standard selective coronary angiography via the radial artery using a 5-F system for coronary anatomy assessment. Intracoronary bolus injection of nitroglycerin (0.2 mg) was administered at the start of the procedure and repeated every 30 min. Fractional flow reserve measurements were obtained using a single 0.014-inch PressureWire™ (Abbott Vascular, St. Paul, MN, USA). Quantitative coronary angiography analyses were performed using a CMS-MEDIS system (Medis Medical Imaging Systems, Leiden, the Netherlands). All patients received a bolus injection of heparin (5000 IU) before the procedure. The type of stent (newer than the second generation drug-eluting stent) for percutaneous coronary intervention was selected at the operator's discretion, and the strategy was determined by the interventionist. Online quantitative coronary angiography was used to help determine the proper stent size to avoid aggressive stent expansion.

CMR Examination

CMR-Acquisition and Cine-CMR

Cardiac gating and heart rate recording were achieved using the vector-cardiogram device. The cine CMR parameters were as follows: repetition time/echo time, 4.1 ms/1.4 ms; slice thickness, 6 mm; flip angle, 55°; field-of-view, 350×350 mm^2; matrix size, 128×128; number of phases per cardiac cycle, 20; and slice thickness, 8 mm. The ventricular volume, ejection fraction, and LV mass were derived by contouring the endo- and epicardial borders on the short-axis cine images. Simpson’s rule was used to calculate the LV mass and volumes using CMR data. LV mass was normalized to body surface area as the LV mass index.

Supplementary Tables

Supplementary Table S1: Baseline patient characteristics divided by the occurrence of primary MACE

	All (n=213)	Patients without primary MACE (n=179)	Patients with primary MACE (n=34)	*P* value
**<u>Baseline data</u>**				
**Demographics**				
Sex				0.509
Men, n (%)	164 (77.0)	136 (76.0)	28 (82.4)	
Women, n (%)	49 (23.0)	43 (24.0)	6 (17.6)	
Age, years	68 [61, 72]	68 [61, 73]	68 [60, 72]	0.683
Body surface area, m^2	1.72 [1.58, 1.82]	1.71 [1.58, 1.82]	1.72 [1.62, 1.84]	0.498
Body mass index, kg m^{-2}	24.4 [22.2, 27.0]	24.1 [22.1, 26.9]	25.4 [22.6, 27.1]	0.266
Rate pressure product at rest	9432 [8100, 10725]	9432 [8208, 10782]	9195 [8006, 9906]	0.367
Rate pressure product at hyperemia	10108 [8585, 11616]	10086 [8493, 11704]	10174 [8810, 10851]	0.720
**Medical history**				
Current smoker, n (%)	53 (24.9)	44 (24.6)	9 (26.5)	0.830
Diabetes mellitus, n (%)	90 (42.3)	69 (38.5)	21 (61.8)	0.014
Hypertension, n (%)	157 (73.7)	132 (73.7)	25 (73.5)	1.000

Hyperlipidemia, n (%)	111 (52.1)	87 (48.6)	24 (70.6)	0.024
Family history, n (%)	28 (13.1)	19 (10.6)	9 (26.5)	0.023
**Laboratory data**				
Creatinine, mg dL^{-1}	0.83 [0.72, 0.95]	0.83 [0.72, 0.95]	0.82 [0.72, 0.93]	0.562
eGFR, ml min^{-1} 1.73m^{-2}	68.6 [58.8, 77.7]	67.9 [57.4, 78.2]	70.9 [63.1, 76.9]	0.256
LDL-C, mg dL^{-1}	97 [75, 120]	93 [74, 121]	106 [94, 118]	0.082
HDL-C, mg dL^{-1}	51 [43, 60]	51 [43, 60]	49 [42, 57]	0.427
Triglyceride, mg/dL	122 [87, 165]	122 [83, 168]	123 [93, 163]	0.934
HbA1c, %	6.1 [5.7, 6.9]	6.0 [5.7, 6.8]	6.9 [6.0, 7.7]	0.006
NT-pro BNP, ng L^{-1}	128 [52, 291]	135 [50, 317]	118 [54, 220]	0.425
hs-cTnI at presentation, ng L^{-1}	4 [2, 11]	7 [3, 21]	12 [5, 30]	0.076
**Angiographic characteristics**				
Lesion location (LAD/LCX/RCA)	140/21/52	120/16/43	9/20/5	0.474
Reference diameter, mm	2.44 [1.98, 2.78]	2.48 [1.98, 2.80]	2.20 [1.95, 2.62]	0.312
Minimum lumen diameter, mm	0.83 [0.58, 1.14]	0.85 [0.58, 1.15]	0.79 [0.52, 1.09]	0.496
Angiographic stenosis severity, %	64.9 [53.1, 76.5]	64.4 [53.4, 76.3]	67.7 [52.2, 76.3]	0.633
SYNTAX score	10.0 [7.0, 15.0]	9.0 [7.0, 14.3]	12.3 [8.3, 16.00]	0.031
pre FFR	0.64 [0.48, 0.73]	0.64 [0.48, 0.73]	0.64 [0.50, 0.73]	0.683

**CMR analysis**				
LV end-diastolic volume, ml	116 [96, 140]	117 [97, 137]	113 [96, 143]	0.909
LV end-systolic volume, ml	41 [31, 60]	41 [31, 61]	42 [31, 58]	0.826
LVEF, %	63 [54, 69]	63 [54, 70]	64 [53, 68]	0.674
LV mass, g	135 [109, 160]	134 [107, 161]	143 [117, 159]	0.114
LV mass index, g m^{-2}	76.5 [65.3, 92.9]	75.5 [64.8, 89.5]	81.1 [70.7, 93.9]	0.113
UMI, n (%)	63 (29.6)	46 (25.7)	17 (50.0)	0.007
LGE mass, g	0.00 [0.00, 4.60]	0.00 [0.00, 2.40]	0.85 [0.00, 11.65]	0.006
**Post PCI data**				
post FFR	0.87 [0.82, 0.92]	0.87 [0.82, 0.92]	0.84 [0.77, 0.90]	0.151
Peak hs-cTnI, ng L^{-1}	229 [82, 1118]	220 [80, 976]	290 [142, 2740]	0.070
Peak CK, IU L^{-1}	104 [73, 187]	102 [69, 178]	112 [86, 233]	0.211
Peak CK-MB, IU L^{-1}	12 [9, 19]	12 [9, 18]	14 [9, 26]	0.319

Abbreviations are in Table 1. primary MACE was defined as cardiovascular death, nonfatal MI, hospitalization for congestive heart failure, unplanned late revascularization, and ischemic stroke.

Supplementary Table S2: The details of the occurrence of MACE in patients with or without UMI

	Total<br>N=213	Patients without UMI<br>N=150	Patients with UMI<br>N=63	P value
Cardiovascular death	1 (0.5)	0 (0.0)	1 (1.6)	0.296
Myocardial infarction	6 (2.8)	1 (0.7)	5 (7.9)	0.009
Heart failure	3 (1.4)	1 (0.7)	2 (3.2)	0.209
Ischemic stroke	3 (1.4)	1 (0.7)	2 (3.2)	0.209
Revascularization	21 (9.9)	14 (9.3)	7 (11.1)	0.802
Composite MACE	34 (16.0)	17 (11.3)	17 (27.0)	0.001

Abbreviations are in Table 1.

Supplementary Figures

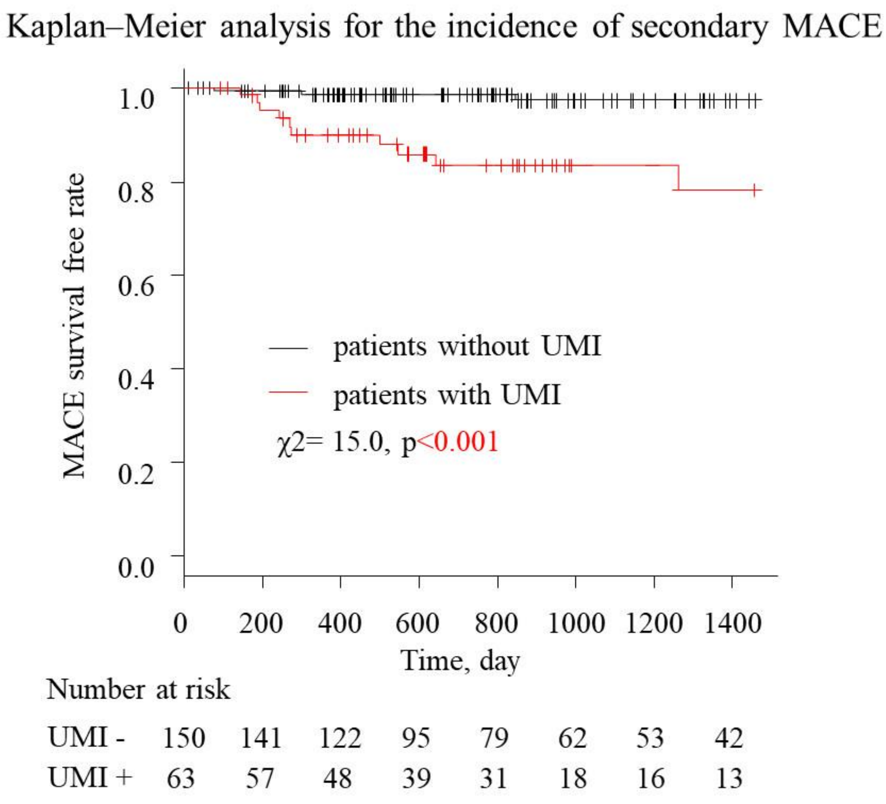

Supplementary Figure S1: Kaplan–Meier analysis for the incidence of secondary MACE according to the presence of UMI

The incidence of secondary MACE was significantly higher in patients with UMI than that in those without UMI. Secondary MACE was defined as cardiovascular death, nonfatal myocardial infarction, hospitalization for congestive heart failure, and ischemic stroke.

Abbreviations: UMI=unrecognized myocardial infarction, MACE=major adverse cardiovascular events

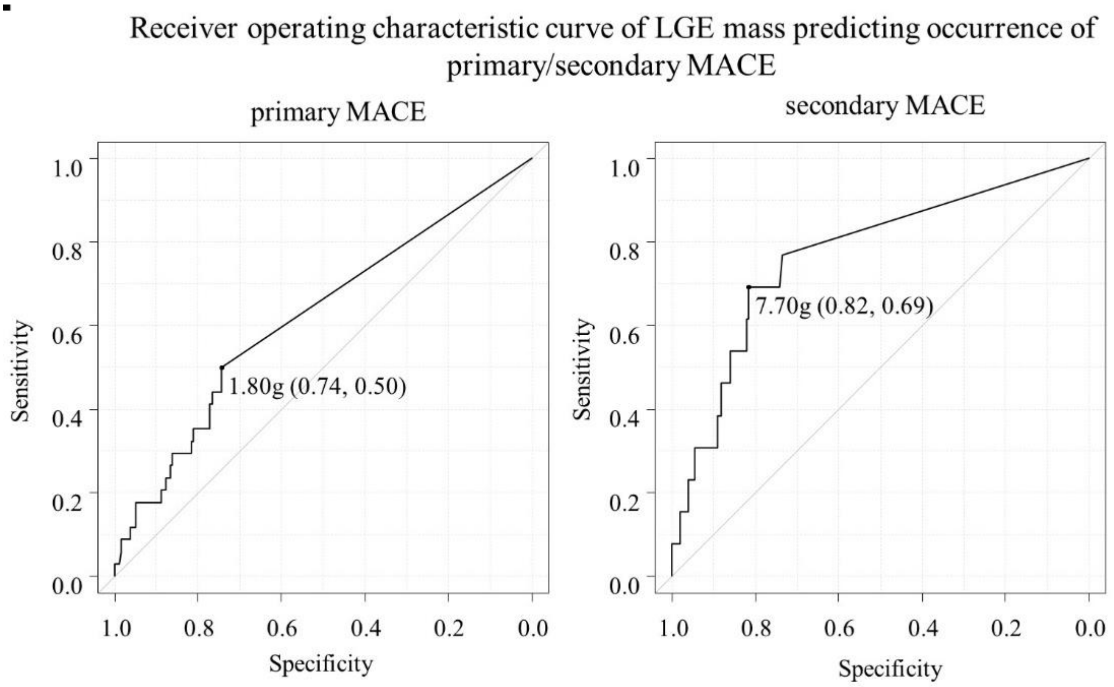

Supplementary Figure S2: Receiver operating characteristic curve for predicting primary and secondary MACE

The best cut-off value of the LGE mass for predicting primary MACE was 1.80 g and the area under the curve was 0.619 (P=0.028). The best cut-off value of the LGE mass for predicting secondary MACE was 7.70 g and the area under the curve was 0.768 (P=0.001).

Abbreviations: MACE=major adverse cardiovascular events; LGE=late gadolinium enhancement
